# High expression of spliced X-Box Binding Protein 1 in lung tumors is associated with cancer aggressiveness and epithelial-to-mesenchymal transition

**DOI:** 10.1038/s41598-020-67243-8

**Published:** 2020-06-23

**Authors:** Quentin Tavernier, Antoine Legras, Audrey Didelot, Corinne Normand, Laure Gibault, Cécile Badoual, Françoise Le Pimpec-Barthes, Pierre Laurent Puig, Hélène Blons, Nicolas Pallet

**Affiliations:** 1 Paris Descartes University, Paris, France; 2 Department of Thoracic Surgery, Georges Pompidou European Hospital, Assistance Publique - Hôpitaux de Paris, Paris, France; 3 Department of Pathology, Georges Pompidou European Hospital, Assistance Publique - Hôpitaux de Paris, Paris, France; 4 Department of Clinical Chemistry, Pharmacogenetics and Molecular Oncology, Georges Pompidou European Hospital, Assistance Publique - Hôpitaux de Paris, Paris, France

**Keywords:** Non-small-cell lung cancer, Lung cancer, Tumour biomarkers, Gene expression profiling, PCR-based techniques, Endoplasmic reticulum, Stress signalling

## Abstract

Proteostasis imbalance is emerging as a major hallmark of cancer, driving tumor growth and aggressiveness. Endoplasmic Reticulum (ER) stress has been documented in most major cancers, and the ability to tolerate persistent ER stress through an effective unfolded protein response enhances cancer cell survival, angiogenesis, metastasis, drug resistance and immunosuppression. The ER stress sensor IRE1α contributes to tumor progression through XBP1 mRNA splicing and regulated IRE1α-dependent decay of mRNA and miRNA. The aim of this study was to perform a molecular characterization of series of tumor samples to explore the impact of intratumoral IRE1 signaling in non-small cell lung cancer characteristics. To monitor IRE1 splicing activity, we adopted a fragment length analysis to detect changes in the length of the XBP1 mRNA before and after splicing as a method for measuring sXBP1 mRNA levels in tumors because sXBP1 mRNA is not probed by standard transcriptomic analyses. We demonstrate for the first time that XBP1 splicing is a valuable marker of lung cancer aggressiveness, and our results support a model in which IRE1 downstream signaling could act as a regulator of Epithelial to Mesenchymal Transition (EMT). Our findings study highlights the role of IRE1α downstream signaling in non-small cell lung cancer and opens a conceptual framework to determine how IRE1α endoribonuclease activity shapes the EMT program.

## Introduction

Multiple cell-intrinsic and cell extrinsic mechanisms within the cancer cell and the tumor microenvironment initiate and amplify proteostasis disturbances within the Endoplasmic Reticulum (ER)^1^. Oncogenic transformation driven by loss of tumor suppressors or activation of oncogenes can lead to dramatic increases in protein synthesis^2–4^, and extrinsic disturbances such as limited nutrients, acidic pH, and hypoxia^5^, lead to an excessive accumulation of misfolded proteins in the ER lumen, a situation called ER stress. Under these conditions, the Unfolded Protein Response (UPR) adapts to the changing environment and reestablishes normal ER function by reducing the amount of newly synthesized proteins in the ER, by increasing the proteasomal degradation of ER-localized proteins, and by increasing the protein-folding capacity of the ER^6^. Three major mediators are involved in UPR response signaling: ATF6 (Activated Transcription Factor 6), IRE1α (Inositol Requiring Enzyme 1α), referred to as IRE1 thereafter, and PERK (Protein Kinase RNA (PKR)-like ER kinase). Insufficient clearance of misfolded proteins results in apoptotic cell death.

There is now clear evidence that ER stress is a hallmark of numerous cancers, and that the UPR can affect tumor cell biology either as a barrier to tumor development, or by promoting established tumors^7–9^. Of particular interest in this process is IRE1, which is critical for cell adaptation capacity under stress conditions, and which has been well documented in its ability to control life or death of tumor cells^10,11^. For example, IRE1 recruits TNF receptor associated factor 2 (TRAF2), and activates c-jun-N-terminal kinase (JNK), which inhibits the anti-apoptotic protein BCL2^12^; conversely, IRE1 facilitates tumor cell growth *in vivo* by promoting the expression of β-catenin^13^. Moreover, IRE1 RNase activity contributes to basal production of pro-tumorigenic factors IL-6, IL-8, CXCL1, GM-CSF, and TGFβ^14^. IRE1 catalyzes the unconventional processing of the mRNA encoding XBP1, and creates a transcriptionally active XBP1 (spliced XBP1, sXBP1) that enters the nucleus. sXBP1 is critical for cellular adaptation in stressful conditions through the control of the expression of genes that encode factors that modulate protein folding, secretion, ER-associated degradation (ERAD), protein translocation into the ER, and lipid synthesis^15^. In addition, active IRE1 RNase is involved in mRNA degradation through Regulated IRE1 Dependent Decay (RIDD), a process critical for ER homeostasis maintenance^11^. Notably, XBP1 mRNA splicing and RIDD can occur simultaneously^16,17^, but can have antagonistic roles on tumor outcomes, where sXBP1 provides pro-tumoral signals and RIDD elicits anti-tumoral features in glioblastoma^16^. Nevertheless, IRE1 emerges as a promising therapeutic target in cancer and numerous IRE1 inhibitors are currently under evaluation^14,18,19^.

A possibility of a role for IRE1 in regulating the Epithelial-to-Mesenchymal Transition (EMT) program is supported by the evidence that sXBP1 drives the expression of several EMT transcription factors including SNAI1, SNAI2, ZEB2 and TCF3^20,21^. Members of the miR-200 family, including miR-200a and miR-429, which plays crucial roles in cancer initiation and metastasis by repressing genes involved in EMT^22–24^, are RIDD targets in bone marrow–derived progenitor cells, and regulate wound healing and angiogenesis^13^.

Whether and how IRE1 activity contributes to Non-Small Cell Lung Cancer (NSCLC) development and progression has attracted little attention, and has been very little explored so far. To address this issue, we performed a molecular characterization of series of tumor samples to explore the impact of intratumoral IRE1 signaling in NSCLC characteristics. We demonstrate that XBP1 splicing is a valuable biomarker of NSCLC aggressiveness, and our results support a model in which IRE1 downstream signaling could act as a regulator of part of the EMT program.

## Patients and Methods

### Patients and tumor samples

One hundred seventy-six (176) primary lung cancer specimens that have been prospectively collected and stored frozen the time of surgery from October 2011 to December 2014, were available for the purpose of the present study. Surgery was performed for curative intent and clinical and survival data were collected in electronic medical records. Of the initial collection of 176 samples, 4 samples corresponding to patients with stage IV NSCLC, and 3 samples with surgically related death were excluded, and 4 samples were excluded for technical reasons. Therefore, 165 subjects were included in the study.

### Reglementary aspects

Biological samples have been provided the Biological Resources center and Tumor Bank Platform (PRB-HEGP BB-0033-00063). Patients involved in the OncoHEGP program signed informed consent for tissue collection and research use. All experimental protocols were approved by the Comité de Protection des Personnes Ile de France 2, n°2012-08-09 and n°2012-08-09 A1. All patients provided written consent for study participation and for the biological analysis before inclusion. The study was performed in accordance with the Declaration of Helsinki.

### Samples preparation

Tumors were cut on a cryostat and reviewed by the pathologist for subsequent DNA and RNA extractions. Mean tumor cells content was 52% +/- 25, 6 samples had less than 20% and 10 were non-tumor samples and excluded. DNA and RNA were extracted using Qiaamp Dna Mini Kit (Qiagen) and Mirneasy Mini Kit (Qiagen) extraction kits, DNAs and RNAs were quantified by Qubit Fluorometric Quantitation (Thermo Fisher Scientific) and stored frozen.

### Mutational analysis

Samples were characterized for molecular alterations by targeted NGS (Ion Ampliseq Colon-Lung Cancer Research Panel V2, Life Technologies). Briefly the multiplex barcoded libraries are generated from 10 ng of DNA following manufacturer’s recommendations (Ion Ampliseq Library Kit V2) and are normalized using the Ion Library Equalizer Kit. The pooled libraries (max 96) are processed on Ion Chef System for template preparation and chip loading (Ion Pi Hi-Q Chef Kit, Ion Pi Chip V3), and sequenced on the Ion Proton System (Life Technologies). The FASTQs sequencing data are aligned to the human genome (hg19) and processed using Iontorrent Suite V5.0.4.0 This package included the Torrent Variant Caller V5.0.4.0 using the built-in “Somatic - low stringency” with optimized parameters to automatically call variants with allelic ratio>2%.

### Fragment analysis of sXBP1 and XBP1 mRNA levels

sXBP1 and XBP1 RNAs were measured by fragment analysis as we previously described^25^. RNA is extracted and reverse-transcribed into cDNAs. The fragment analysis involves 3 steps: (1) sXBP1 and XBP1 cDNAs are amplified by PCR using fluorogenic oligonucleotide primers (tagged with hexachloro-fluorescein, HEX, green): Forward primer: (5’-HEX)GGAGTTAAGACAGCGCTTGG and Reverse Primer: GAGATGTTCTGGAGGGGTGA. PCR has been performed using HotStar Taq DNA polymerase on a thermal cycler with the following program: 95 °C for 10 min; 40 cycles of 94 °C for 30 sec, 59 °C for 30 sec, and 72 °C for 30 sec; and a final step of 72 °C for 10 min. (2) Labeled fragments (amplicons) are separated by size using capillary electrophoresis and the fluorescence intensity is measured using the Applied Biosystems 3730xl DNA Analyzer. One of the dye colors (Genescan Rox 400 Hd size standard, Applied Biosystems, red) is used to detect a labeled size standard present in each sample. Fragments and ROX 400 HD are mixed with HiDi Formamide (Applied Biosystems) prior to capillary electrophoresis. (3) The data are analyzed using Genemapper Software to determine the relative size of each dye‐labeled fragment in the sample.

### EMT genes expression and scoring system

Real-time quantitative polymerase chain reaction (qPCRs) were performed to characterize EMT and stem cell markers in tumors, using 5 mesenchymal markers (TWIST1, N-CADHERIN, ZEB1, SNAI1, VIMENTIN), 2 epithelial markers (E-CADHERIN, JUP) and 2 stem cells markers (TCF3, CD44). RNAs (1 μg) were reversed-transcribed using the High Capacity cDNA Reverse Transcription Kit (Applied Biosystem). E-CADHERIN, JUP, TWIST1, N-CADHERIN, ZEB1, SNAI1, VIMENTIN, TCF3 and CD44 mRNA levels were quantified using FAM Taqman probes on an ABI Prism 7900 sequence detector system (TaqMan Gene Expression Assay, Applied Biosystems). Measures were performed in triplicate with 10 ng/μL of cDNA using Taqman Gene Expression Master Mix (Applied Biosystems). We performed duplex analysis using 18 S RNAs (VIC probe, Applied Biosystems) as endogenous gene control. Low quality RNA samples (18 S RNA Ct>16) were discarded from the analysis. Expression levels were calculated as described by Livak and Schmittgen. Fold change defined down, stable or up-regulation of the quantified markers.

For each sample, the fold change of a defined EMT marker was categorized as:<1 indicated down regulation, 1-2 indicated stable expression, and>2 indicated up-regulation. Fold changes of each EMT marker were calculated in using the 2^-ΔΔCT^ method^26^ using 18 S RNA as internal control, and a pool of RNA normal lung tissues (n = 10) prepared in our laboratory, as a calibrator. As we quantified 2 epithelial markers and 5 mesenchymal markers, we weighted results to create an EMT score to classify tumors: for epithelial markers, down, stable and up-regulation were weighted as 10, 5 and 0 points; for mesenchymal markers, down, stable and up-regulation were weighted as 0, 2 and 6 points. Low EMT score indicates an epithelial phenotype and high EMT score, a mesenchymal phenotype. This classification was established a priori.

### MicroRNA expression

qPCRs were performed to investigate expression levels of miR-200a-3p in this series. RNAs (10 ng/μL) were reversed-transcribed using the TaqMan advanced miRNA cDNA Synthesis Kit (Applied Biosystem). MiRs levels were quantified by qPCR using Taqman probes (Taqman Advanced Mirna Assay, clone ID 478490_mir, Thermo Fisher Scientific), on an ABI Prism 7900 system (Applied Biosystems). Real-time PCR was performed in triplicate with 3.3 ng of cDNA and the Taqman Fast Advanced Master Mix (Applied Biosystems). MiR-423 and miR-425 (clone ID 478327_mir and clone ID 478094_mir, Thermo Fisher Scientific) were used for normalization and expression levels were calculated as 2^−ΔCT^ described by Livak and Schmittgen^27^. In each run, normal human lung RNA as described above was used as calibrator.

### Intratumor CHOP transcripts expression

CHOP Transcripts were quantified using SYBR green (CHOP) on an ABI Prism 7900 sequence detector system (Applied Biosystems). Measures were made in triplicates with 10 ng/μl of cDNA using Taqman Gene Expression Assay Master Mix (Applied Biosystems). Real-time PCR data for CHOP were presented as individual data points, and reported as expression levels (cycle thresholds, Ct) referred to as an internal control. Therefore, Ct are calculated as (Ct gene of interest - Ct internal control). Transcripts expression levels were presented as 2^−ΔCT^ as recommended by^27^.

### Statistical analysis

Continuous variables are presented as the mean and standard deviation and categorical variables are presented as presented as proportions. For variance analysis of continuous variables in different groups, Student’s test and one-way ANOVA were performed to compare two or more groups. The rate of disease-free survival was calculated using Kaplan-Meier curves, and statistical comparisons between survival distributions were made using the Wilcoxon test. Pearson’s correlation coefficients were calculated to evaluate linear correlation between the distributions of 2 continuous variables. For assessing the determinants of EMT class, multiple linear regression model (standard least squares) and nominal logistic regression analysis were performed, respectively. Statistical analyses were performed using JMP.10 (Sas Software). All tests were two-sided, and *P* values <0.05 were considered to indicate significance.

## Results

### The IRE1-sXBP1 pathway is activated NSCLC and correlate with tumor aggressiveness

To monitor IRE1 splicing activity, we developed a method for measuring sXBP1 mRNA levels in tumors. Indeed, sXBP1 mRNA is not probed by standard transcriptomic analyses. Because the XBP1 mRNA is 26 nucleotides longer than sXBP1, we adopted a fragment length analysis to detect changes in the length of the XBP1 mRNA before and after splicing. The PCR amplicons (fragments) are separated according to their sizes and analyzed on a capillary electrophoresis-based DNA sequencing instrument^25^ (see **Methods** and Fig. S1). Tumor samples retrieved after surgical resection in a cohort of 165 subjects with NSCLC prospectively followed at the Georges Pompidou European hospital were examined. In addition, we measured the expression of CHOP, the key regulator of apoptosis upon ER stress, within tumors, by RT-qPCR. Since CHOP expression depends on PERK activity, and is independent of IRE1 activity, it serves as a control to assess the specificity of IRE1 activity in relation to ER stress in this setting. The clinical and tumor parameters are listed in the Table 1.

**Table 1:** Clinical characteristics of the cohort of patients with NSCLC.

Characteristic	Entire cohort (n = 165)
Age (years)	66±11.3
Sex ratio M/F	104/50
IMC (kg/m^2^)	24.5±4.2
ASA -n(%)	
score 1	11 (8)
score 2	82 (59)
score 3	44 (32)
OMS-n(%)	
score 0	40 (29)
score 1	80 (59)
score 2	13 (9.7)
score 3	1 (0.7)
Type-n(%)	
Adenocarcinoma	105 (68)
Squanmous cell carcinoma	38 (25)
Large cell carcinoma	7 (4.5)
Carcino-sarcoma	4 (2.5)
Stage-n(%)	
I	70 (45)
II	41 (26)
III	43 (28)
Neoadjuvant chemotherapy-n(%)	16 (10)
Adjuvant chemotherapy-n(%)	59 (38)
Adjuvant radiotherapy-n(%)	24 (15)
Mutations-n(%)	
*TP53*	64 (41)
*KRAS*	45 (29)
*EGFR*	17 (11)
*STK11*	8 (5)
*PI3KCA*	6 (4)
*BRAF*	4 (2.5)
*NRAS*	3 (2)
*CTNNB1*	3 (2)
*SMAD4*	2 (1)
*FBXW7*	1 (0.5)
*ERBB2*	2 (1)
*AKT1*	2 (1)
*ERBB4*	3 (2)
*FGFR3*	1 (0.5)
*PTEN*	2 (2)
*DDR2*	1 (0.5)
*MAP2K1*	1 (0.5)

**Figure 1:**
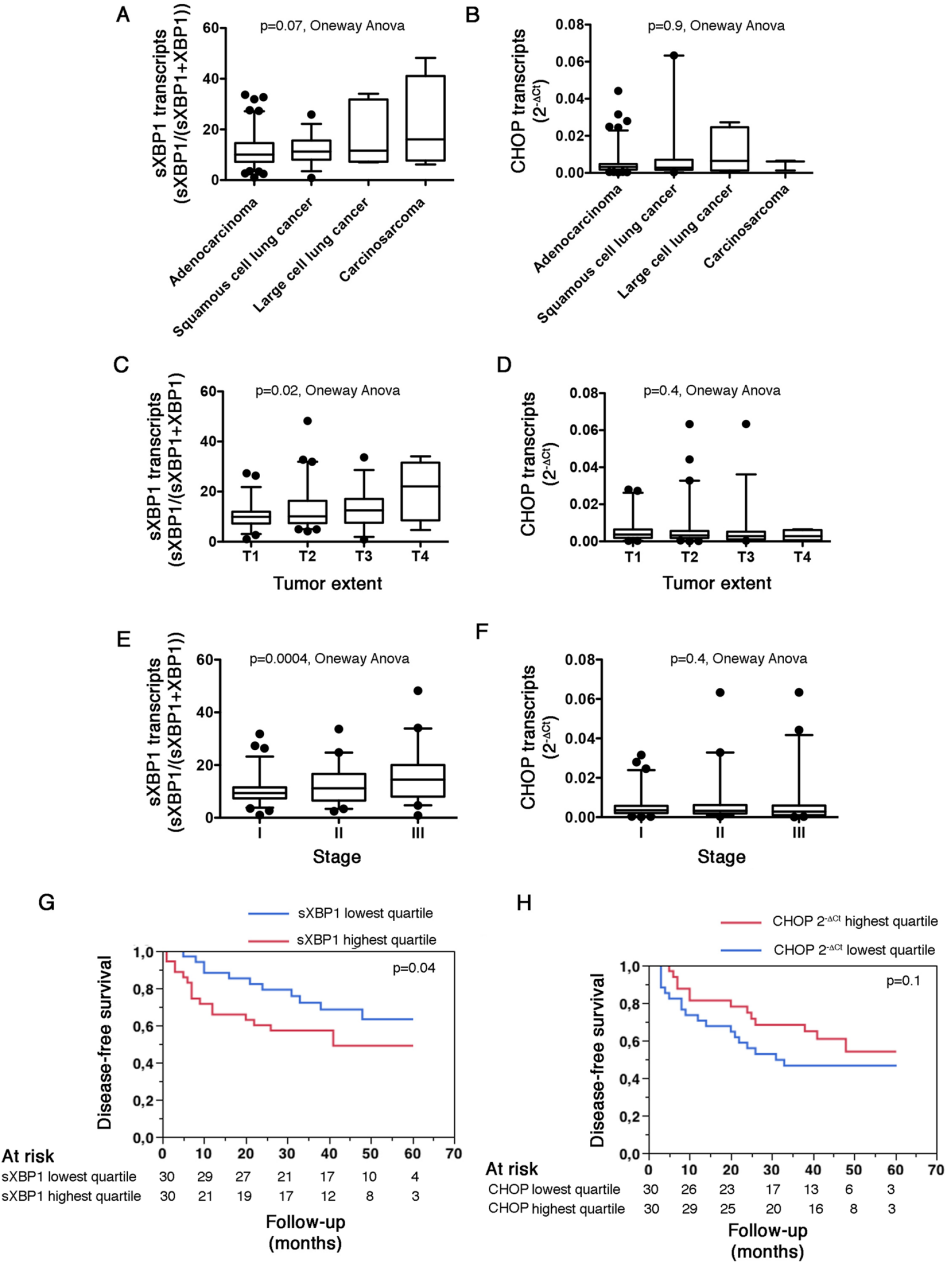
The IRE1-sXBP1 pathway is activated NSCLC and correlate with tumor aggressiveness. **A**. Box and whiskers plots representing the amount of sXBP1 transcripts measured by fragments analysis in NSCLC tumor samples, classified according to the histological type of cancer. sXBP1 expression is calculated as sXBP1 peak intensity/(sXBP1 + XBP1 peak intensity). Oneway analysis of variance. **B**. Box and whiskers plots representing the amount of CHOP transcripts measured by RT-qPCR in NSCLC tumor samples, classified according to the histological type of cancer. CHOP relative expression levels are measured as cycle thresholds normalized to endogenous gene control (Ct). Oneway analysis of variance. **C**. Box and whiskers plots representing the amount of sXBP1 transcripts measured by fragments analysis in NSCLC tumor samples, classified according to the size and extend of the tumor. sXBP1 expression is calculated as sXBP1 peak intensity/(sXBP1 + XBP1 peak intensity). Oneway analysis of variance. **D**. Box and whiskers plots representing the amount of CHOP transcripts measured by RT-qPCR in tumor samples, classified according to the stage of the tumor. CHOP relative expression levels are measured as cycle thresholds normalized to endogenous gene control (Ct). Oneway analysis of variance. **E**. Box and whiskers plots representing the amount of sXBP1 transcripts measured by fragments analysis in NSCLC tumor samples, classified according to the stage of the tumor. sXBP1 expression is calculated as sXBP1 peak intensity/(sXBP1 + XBP1 peak intensity). Oneway analysis of variance. **F**. Box and whiskers plots representing the amount of CHOP transcripts measured by RT-qPCR in tumor samples, classified according to the size and extend of the tumor. CHOP relative expression levels are measured as cycle thresholds normalized to endogenous gene control (Ct). Oneway analysis of variance. **G**. Kaplan-Meier curves for the association between the amount of sXBP1 transcripts in tumor samples and disease-free survival in the cohort of 165 patients with NSCLC, according to highest (red curve) and lower (bleu curve) quartiles of sXPB1 distribution, measured as sXBP1 peak intensity/(sXBP1 + XBP1 peaks intensity). Wilcoxon test. **H**. Kaplan-Meier curves for the association between the amount of CHOP transcripts in tumor samples and disease-free survival in the cohort of 165 patients with NSCLC, according to highest (red curve) and lower (bleu curve) quartiles of CHOP distribution. Wilcoxon test.

Intra tumor sXBP1 levels are not influenced by the histological type of cancer, albeit they are higher in carcino-sarcoma, made of a mixture of carcinomatous and sarcomatous elements, than in the other types of lung cancers (Fig. 1A). In addition, the histological type of cancer did not impact intra tumor CHOP levels (Fig. 1B). We found a positive association between sXBP1 transcripts (and not CHOP transcripts) with the size and extent of the tumor (Fig. 1C,D) and the stage (Fig. 1E,F) of the cancer, indicating that the activity of the IRE1-XBP1 axis increases in parallel to the growth of the tumor. The unadjusted disease-free survival of individuals in the highest quartile of sXBP1 transcripts was lower compared with those in the lowest quartile, in the whole cohort, and in subjects with adenocarcinoma (Fig. 1G and S2A), whereas the disease-free survival of patients with tumors expressing the highest amount of CHOP transcripts was similar to those with the lowest levels **(**Fig. 1H**)**, and the disease-free survival of individuals in the highest quartile of CHOP transcripts was higher compared with those in the lowest quartile in subjects with adenocarcinoma, which is consistent with the pro-apoptotic functions of CHOP (Fig. S2B**)**. However, after adjustment for the tumor stage, the effect of sXBP1 on survival was no longer significant (not shown). Together, these data indicate that the IRE1-XBP1 axis, rather than the PERK-CHOP axis, is activated in NSCLC, is associated with tumor growth, and may affect tumor aggressiveness.

### Activity of the IRE1-sXBP1 pathway is not related to driver mutations

**Figure 2:**
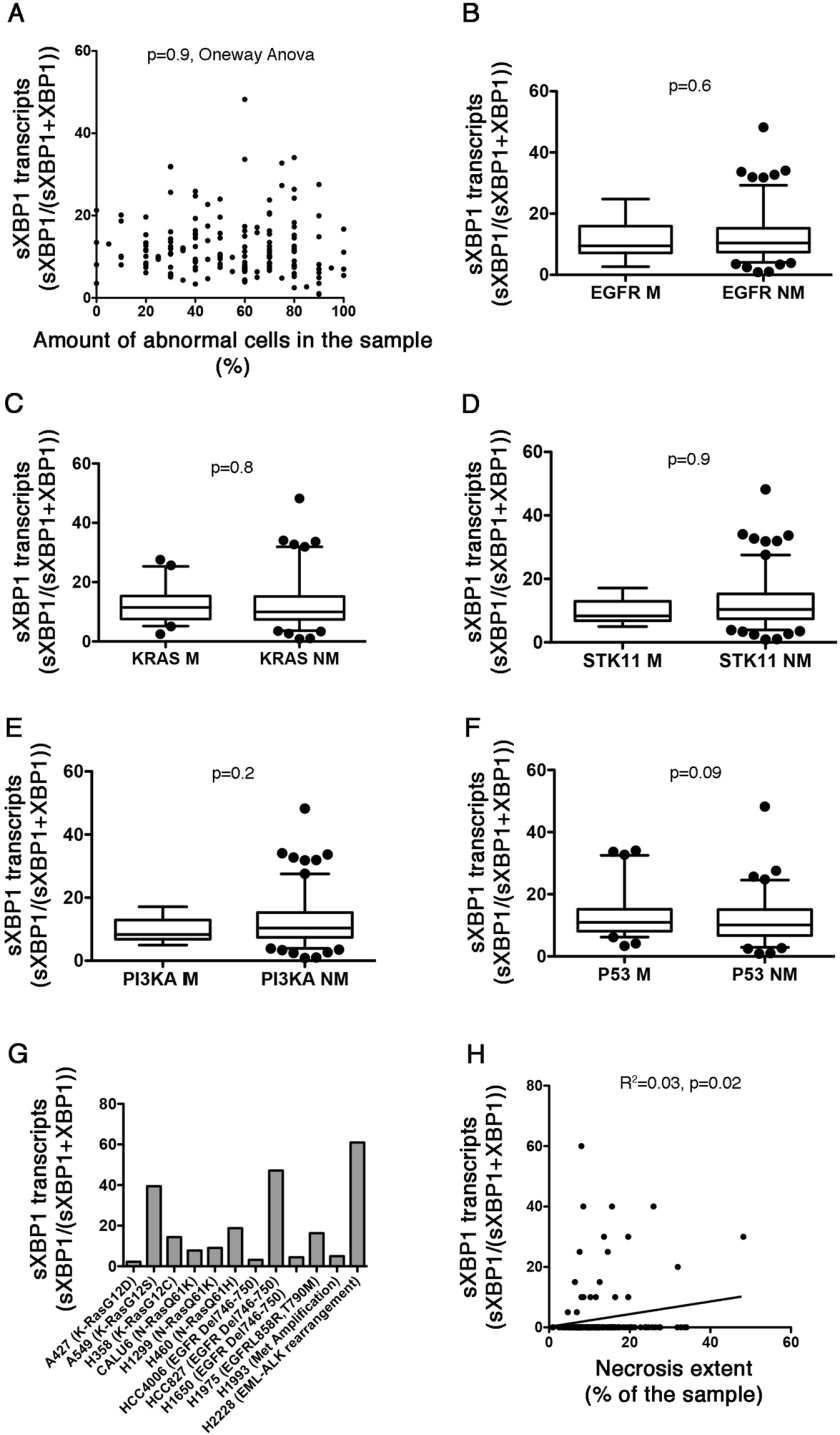
Activity of the IRE1-sXBP1 pathway is not oncogene-driven. **A**. Distribution of the amount of sXBP1 transcripts according to the percentage of cancer cells in the corresponding tumor sample. Oneway Anova. **B**. Box and whiskers plots representing the distribution of the amount of sXBP1 transcripts measured by fragments analysis in tumor samples, according to the presence (M) or absence (NM) of mutation in the gene encoding EGFR. Student’s T test. **C**. Box and whiskers plots representing the distribution of the amount of sXBP1 transcripts measured by fragments analysis in tumor samples, according to the presence (M) or absence (NM) of mutation in the gene encoding KRAS. Student’s T test. **D**. Box and whiskers plots representing the distribution of the amount of sXBP1 transcripts measured by fragments analysis in tumor samples, according to the presence (M) or absence (NM) of mutation in the gene encoding STK11. Student’s T test. **E**. Box and whiskers plots representing distribution of the amount of sXBP1 transcripts measured by fragments analysis in tumor samples, according to the presence (M) or absence (NM) of mutation in the gene encoding PI3KA. Student’s T test. **F**. Box and whiskers plots representing distribution of the amount of sXBP1 transcripts measured by fragments analysis in tumor samples, according to the presence (M) or absence (NM) of mutation in the gene encoding P53. Student’s T test. **G**. A representative histogram representing the amount of sXBP1 transcripts in 12 NSCLC cell lines. Oncogenic driver mutations associated with these cell lines is indicated. **H**. Linear regression curve between the amount of sXBP1 transcripts levels and the proportion of necrotic tissue in the corresponding tumor.

We next sought to determine which mechanism, between tumor microenvironment and oncogenic mutations may initiate IRE1 activity within the cancer cell^1,28^. The intra-tumor amount of sXBP1 transcripts was not influenced by the amount of abnormal cells within the sample, and was even similar to the samples of tumoral tissue without abnormal cells (Fig. 2A), which pleads against a cell-intrinsic mechanism of activation. In addition, there was no difference in the levels of sXBP1 transcripts according to the mutational status of validated oncogenic drivers in the tumor sample (*KRAS* and *EGFR*), potential oncogenic drivers (*STK11* and *PI3KCA*) and other mutated genes, including *P53* (Fig. 2B–F). Consistent with this, XBP1-splicing activity of IRE1 in 10 NSCLC cell lines was not influenced by the presence of a mutation in the oncogenic drivers *KRAS* and *EGFR* (Fig. 2G). The highest basal expression of sXBP1 in these cell lines was found in H2228 cells carrying the *EML4-ALK* fusion gene, which warrants further investigations. Notably, PI3K and KRAS mutations are associated with lower expression levels of CHOP (Fig. S3). Finally, the amount of sXBP1 transcripts, but not CHOP, was positively correlated with the extent of necrotic tissue within the tumor (Fig. 2H and Fig. S3), which can reflect the impact of hostile tumor microenvironmental conditions on the activity of the IRE1-sXBP1 axis. Together, these results indicate that the IRE1-sXBP1 axis is activated in NSCLC most likely in response to harsh extracellular conditions that promote ER stress, rather than to cell-intrinsic oncogenic mutations.

### Association of sXBP1 with the EMT program in NSCLC

**Figure 3:**
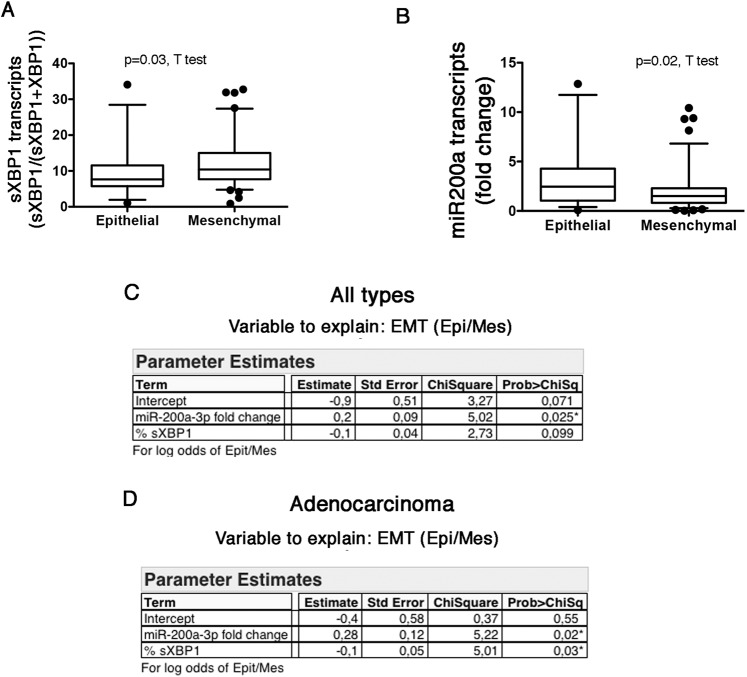
*Association of sXBP1 with the EMT program in NSCLC*. **A**. Box and whiskers plots representing distribution of the amount of sXBP1 transcripts measured by fragments analysis in tumor samples, according to the EMT class (epithelial or mesenchymal). **B**. Box and whiskers plots representing distribution of the amount of miR200a transcripts measured by fragments analysis in tumor samples, according to the EMT class (epithelial or mesenchymal). **C**. Multiple logistic regression analysis between EMT class (Epithelial/Mesenchymal) and miR-200a and sXBP1 as explanatory variables in all the types of cancer. **D**. Multiple logistic regression analysis between EMT class (Epithelial/Mesenchymal) and miR-200a and sXBP1 as explanatory variables in adenocarcinoma.

The UPR can contribute to multiple steps along the EMT program, and the IRE1-sXBP1 axis can regulate the expression of EMT transcription factors^20,29–31^. Then, we tested whether IRE1 activity can be involved in shaping part of the EMT program in NSCLC. We developed an EMT classifier based on the fold change of epithelial markers CDH1 (encoding E-cadherin) and JUP (encoding plakoglobin), and mesenchymal markers including SNAI1 (encoding snail homolog 1), TWIST1 (encoding twist-related protein1), CDH2 (encoding N-cadherin), ZEB1 (encoding zinc-finger E-box binding homeobox 1), and VIM (encoding Vimentin), allowing us to classify tumor samples as “Epithelial” or “Mesenchymal” according to their EMT score (see **Methods**). The amount of intra-tumor sXBP1 transcripts was significantly higher in tumors classified as “Mesenchymal” compared with those classified as “Epithelial”, whereas CHOP transcripts were similar between the 2 groups, (Fig. 3A and S4), suggesting that sXBP1 is associated with the transition to a mesenchymal phenotype. This indicates that the IRE1-sXBP1 axis likely participates in part of the EMT program in NSCLC. The fact that CHOP is not associated with the EMT phenotype argues against a non-specific effect of cellular ER stress on EMT.

IRE1 can degrade miR-200a through RIDD^13^, which could lead to the de-repression of their target EMT transcription factors^23,24^. As expected, the amount of miR-200a transcripts in the tumors was significantly lower in tumors classified as “Mesenchymal” compared with those classified as “Epithelial” (Fig. 3B**)**, which is consistent with the effects of this miRNA on the EMT programm^23,24^. Since sXBP1 and miR-200a potentially drive EMT, we performed a multivariate analysis to model EMT status (Epithelial versus Mesenchymal), and that included sXBP1 and miR200a as explanatory variables, to determine which of these mediators are independent predictors of the EMT status in NSCLC. In the whole cohort, only miR200a transcripts levels remained independently correlated with the EMT status **(**Fig. 3C**)**. However, in the subgroup of subjects with adenocarcinoma (n = 105), sXBP1 and miR200a transcripts levels were independent predictors of the EMT phenotype (Fig. 3D**)**. Together, these results suggest that in NSCLC, and in particular in adenocarcinoma, sXBP1 and miR-200a may independently regulate EMT.

## Discussion

Our work demonstrates for the first time that in a cohort of patients with NSCLC, intra-tumor IRE1 splicing activity of XBP1 mRNA may impact tumor phenotypes and outcomes. Therefore, monitoring intra-tumor IRE1 activity appears as a valuable biomarker for predicting cancer aggressiveness, and could help tailoring pharmacological strategies targeting IRE1 activity. Indeed, several functional studies have shown that targeting the expression or the RNase activity of IRE1 reduces the progression of various forms of cancer mostly due to ablating the prosurvival effects of XBP1 on tumor growth^9,14,32^. Importantly, we provide evidence that measuring the levels of sXBP1 transcripts in tumors by using a fully automatable genetic method based on fragment analysis is clinically relevant, this especially as the expression of sXBP1 is not easily captured by classical transcriptomic platforms for technical reasons (except maybe in public RNAseq datasets using high read depths), and, therefore, the clinical relevance of XBP1 splicing in cancer development and progression has not been specifically addressed. Thus, our method allows to directly monitoring XBP1 splicing activity, and might reflect activation of the IRE1/XBP1 axis in tumors with a lot more simplicity than the analysis sXBP1 target genes signatures^3^.

Previous studies demonstrated the importance of IRE1 signaling for tumor aggressiveness^9,16,32^, but its impact on NSCLC, one of the most frequent cancer worldwide, development and progression, has not been comprehensively examined to date. At first, our clinical and *in vitro* results indicate that the NSCLC mutations P53, KRAS, STK11, EGFR, PI3KCA are not directly involved in the activation of the UPR and the IRE1-sXBP1 axis, while it has been shown that loss of tumor suppressors such as P53^2^ or overexpression of oncogenic HRAS or BRAF^3,33^ enhance protein translation and synthesis, a key driver of ER stress and tumorigenicity^4,34^. However, oncogene expression does not always induce ER stress, and the link between oncogenic mutations and UPR activation is likely a multilayered process involved drivers of protein synthesis such as Myc^35^. Interestingly, we observed a large variation in expression of sXBP1 and CHOP across the lung cancer cell lines we have tested, which supports the possibility that other cell intrinsic activators, such as copy-number variation, epigenetic modifications, or miRNA-mediated regulatory mechanisms, may promote ER stress trough intense increases in protein synthesis. In particular, non-synonymous, non-driver mutations that may affect protein folding, and which are numerous in lung cancer^36^, may induce ER stress by overwhelming ER-folding capacity.

Our results highlight the crosstalk between IRE1 activity and EMT program. EMT is a critical driver for cancer aggressiveness, but the implication of the UPR in regulating the EMT program is only emerging. sXBP1 drives the expression of several EMT transcription factors including SNAI1, SNAI2, ZEB2 and TCF3^20,21^. In addition, PERK enhances cell migration and invasion *in vitro* and *in vivo*, and ATF4 target gene expression correlates with EMT gene signature in cancers including lung cancer^37^. Moreover, PERK is a potential therapeutic target to reduce EMT and invasiveness since PERK inhibition reduces lung metastasis^29,30^. The identification, location and quantification of mesenchymal cells within the tumor, as well as the strength of the correlation could further exemplify the relationship between the IRE1-splicing activity and the activation of the EMT program. As ER stress is a common feature of aggressive cancer, understanding how the UPR modulates disease is critical for identifying promising new clinical strategies. The cardinal features of the consequences of sustained ER stress in cancer progression are the promotion of cell survival, metastasis and invasiveness, angiogenesis, and the promotion of a tolerogenic microenvironment^1^. Critically, IRE1 drives all of these processes, which make it a highly relevant therapeutic target. It is likely that inhibition of IRE1 Kinase or endoribonuclease with small molecules^19^ or selective inhibition of the XBP1 mRNA ligase RtcB^38^, might lead to significant impairment of tumor growth. Notably, numerous compounds targeting IRE1 endonuclease domain (toyocamycin, STF-083010, 4μ8 C, MKC-3946, and B-I09) are currently evaluated in preclinical models of ER stress related disease, including cancer, with promising results^3,39,1^. However, it remains to be determined whether treatment with IRE1 inhibitors recapitulates IRE1 genetic ablation and limits EMT. These therapeutic potentials support the importance of intratumoral ER stress monitoring, which may be a valuable biomarker for determining whether to use UPR-activating or inhibiting compounds.

Collectively, our work demonstrates for the first time that the signals downstream of IRE1 may impact NSCLC progression, and points toward an alternative therapeutic avenue coupled with personalized molecular diagnosis for decreasing tumor cells’ adaptive properties, and modulating the tumor aggressiveness through reduced EMT.

## Supplementary information

Supplementary information.

Supplementary information2.

Supplementary information3.

Supplementary information4.

Supplementary information5.
