# Supplementary material for: High expression of spliced X-Box Binding Protein 1 in lung tumors is associated with cancer aggressiveness and epithelial-to-mesenchymal transition
Source: Sci Rep. 2020 Jun 23;10:10188. doi: 10.1038/s41598-020-67243-8 (PMC7311525; doi:10.1038/s41598-020-67243-8)
Supplement: Supplementary file 1 — Supplementary information. [file 41598_2020_67243_MOESM1_ESM.docx]

**Supplementary figures legends**

**Figure S1.**

**A.** The pattern of peaks resulting from the fragment analysis using capillary electrophoresis with GeneMapper©. Green peaks denote XBP1 (166 nucleotides) and sXBP1 (140 nucleotides) amplicons. The height of each peak corresponds to its relative fluorescence intensity. The x-axis denotes the fragment size. The pattern of peaks resulting from the fragment analysis performed on mRNA extracted from tumor samples, representative of the absence (upper panel), mild (middle panel) of high (lower panel) splicing of XBP1.

**B.** Total mRNAs were extracted from cultured H1650 and H2228 cell lines that had been incubated with 2.5 μM tunicamycin for 4 hours to induce ER stress, and with or without 4μ8C to inhibit IRE1 endoribonuclease activity.

**Figure S2.**

**A.** Kaplan-Meier curves for the association between the amount of sXBP1 transcripts and disease-free survival in the cohort of 105 patients with Adenocarcinoma, according to highest (red curve) and lower (blue curve) quartiles of sXPB1 levels, measured as sXBP1 peak intensity/(sXBP1 + XBP1 peak intensity). Wilcoxon test.

**B.** Kaplan-Meier curves for the association between the amount of sXBP1 transcripts and disease-free survival in the cohort of 105 patients with NSCLC, according to highest (red curve) and lower (blue curve) quartiles of CHOP levels, measured as 2^-ΔCT.^ Wilcoxon test.

**Figure S3.**

**A.** Distribution of the amount of CHOP transcripts according to the percentage of cancer cells in the corresponding tumor sample.

**B.** Box and whiskers plots representing the distribution of the amount of CHOP transcripts according to the presence (M) or absence (NM) of mutation in the gene encoding EGFR. Student’s T test.

**C.** Box and whiskers plots representing the distribution of the amount of CHOP transcripts according to the presence (M) or absence (NM) of mutation in the gene encoding KRAS. Student’s T test

**D.** Box and whiskers plots representing the distribution of the amount of CHOP transcripts according to the presence (M) or absence (NM) of mutation in the gene encoding STK11. Student’s T test

**E.** Box and whiskers plots representing the distribution of the amount of CHOP transcripts according to the presence (M) or absence (NM) of mutation in the gene encoding PI3KA. Student’s T test

**F.** Box and whiskers plots representing the distribution of the amount of CHOP transcripts according to the presence (M) or absence (NM) of mutation in the gene encoding P53. Student’s T test

**G.** Histograms representing the amount of CHOP transcripts in 12 NSCLC cell lines. Oncogenic driver mutations associated with these cell lines is indicated.

**H.** Linear correlation curve between the amount of CHOP transcripts and the proportion of necrotic tissue in the corresponding tumor.

**Figure S4.**

Box and whiskers plots representing distribution of the amount of CHOP transcripts in tumor samples, according to the EMT status (Epithelial/Mesenchymal).
