# Supplementary figures and images for: High expression of spliced X-Box Binding Protein 1 in lung tumors is associated with cancer aggressiveness and epithelial-to-mesenchymal transition
Source: Sci Rep. 2020 Jun 23;10:10188. doi: 10.1038/s41598-020-67243-8 (PMC7311525; doi:10.1038/s41598-020-67243-8)

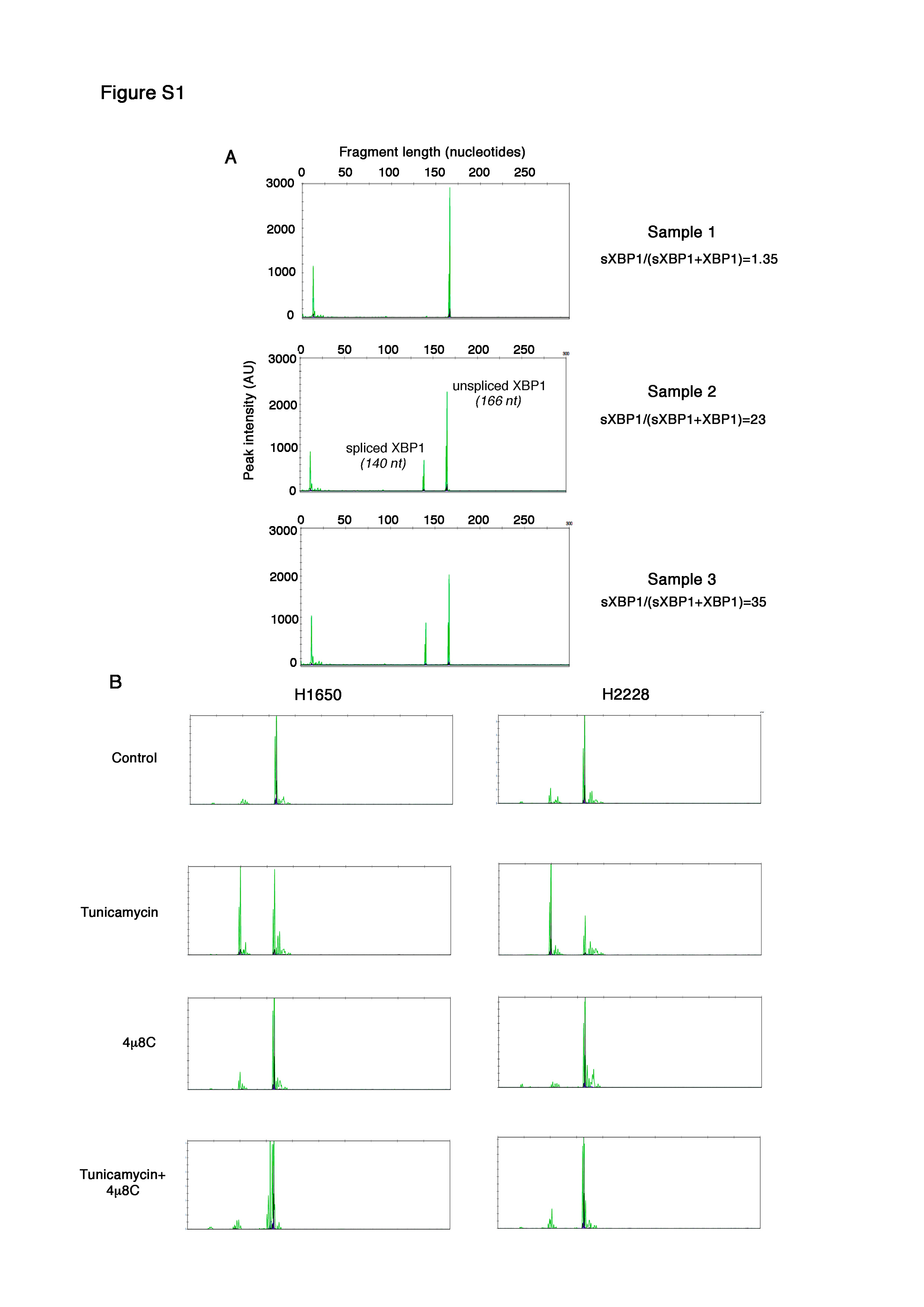

Supplement: Supplementary file 2 — Supplementary information2. [file 41598_2020_67243_MOESM2_ESM.tif]

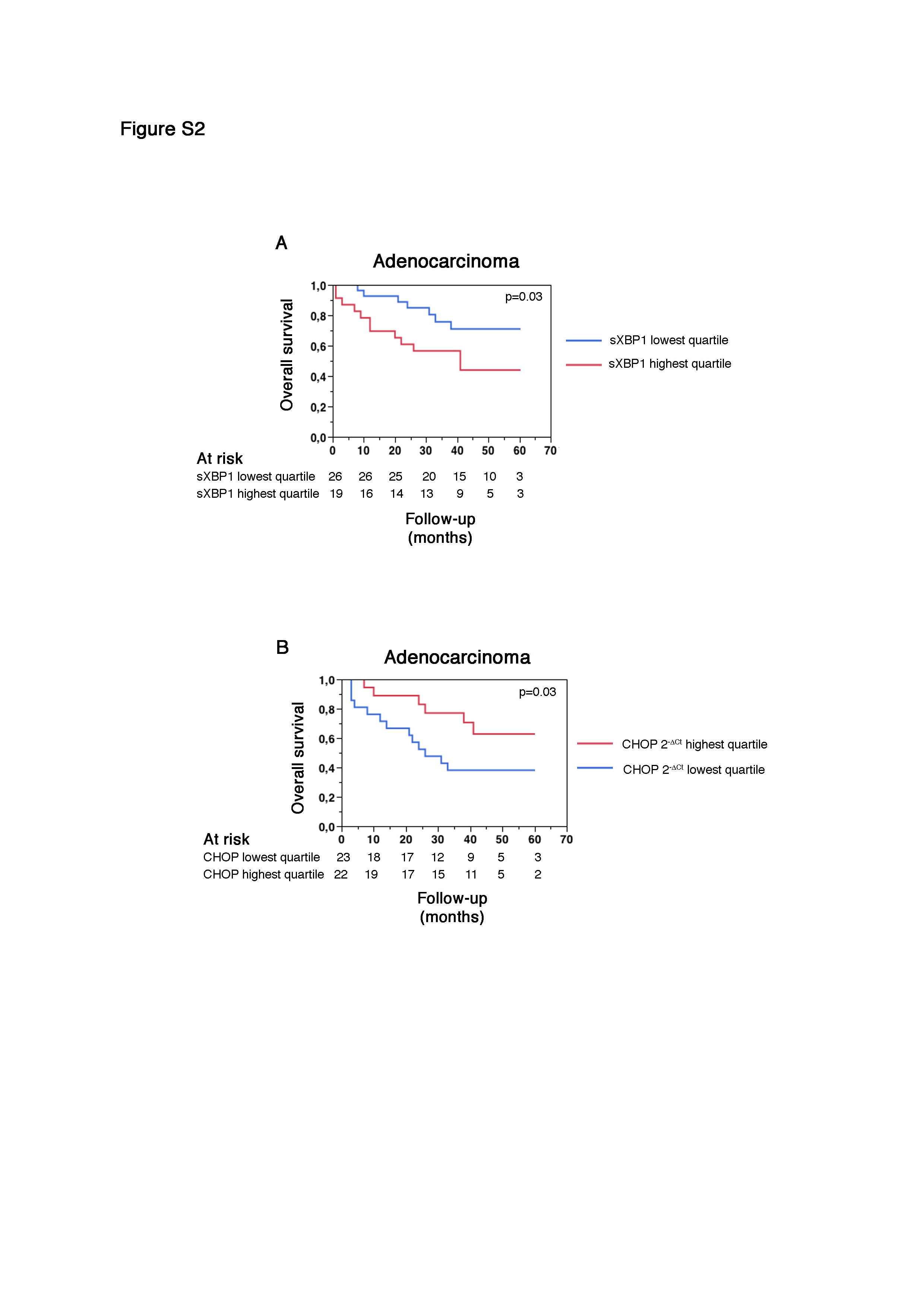

Supplement: Supplementary file 3 — Supplementary information3. [file 41598_2020_67243_MOESM3_ESM.tif]

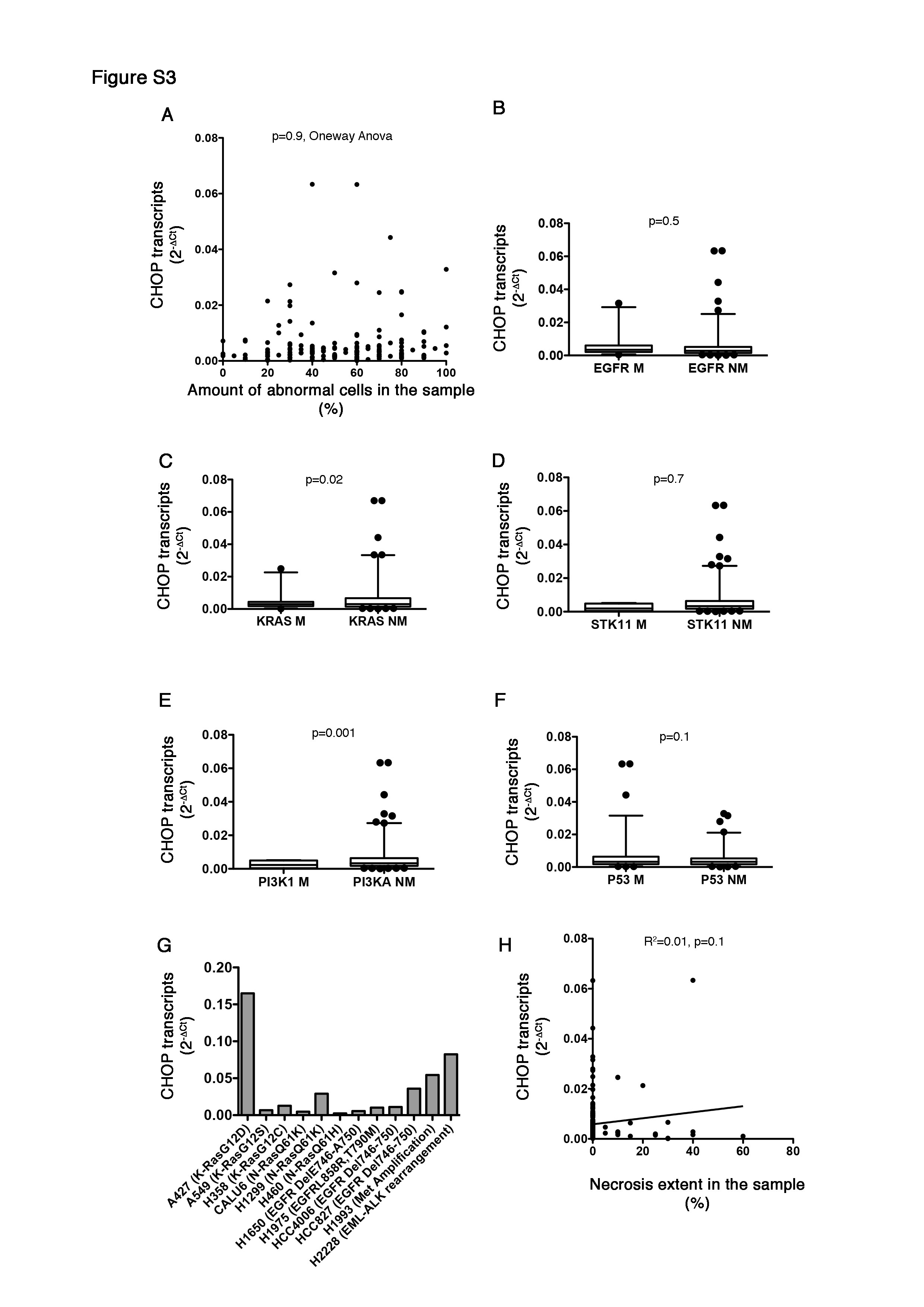

Supplement: Supplementary file 4 — Supplementary information4. [file 41598_2020_67243_MOESM4_ESM.tif]

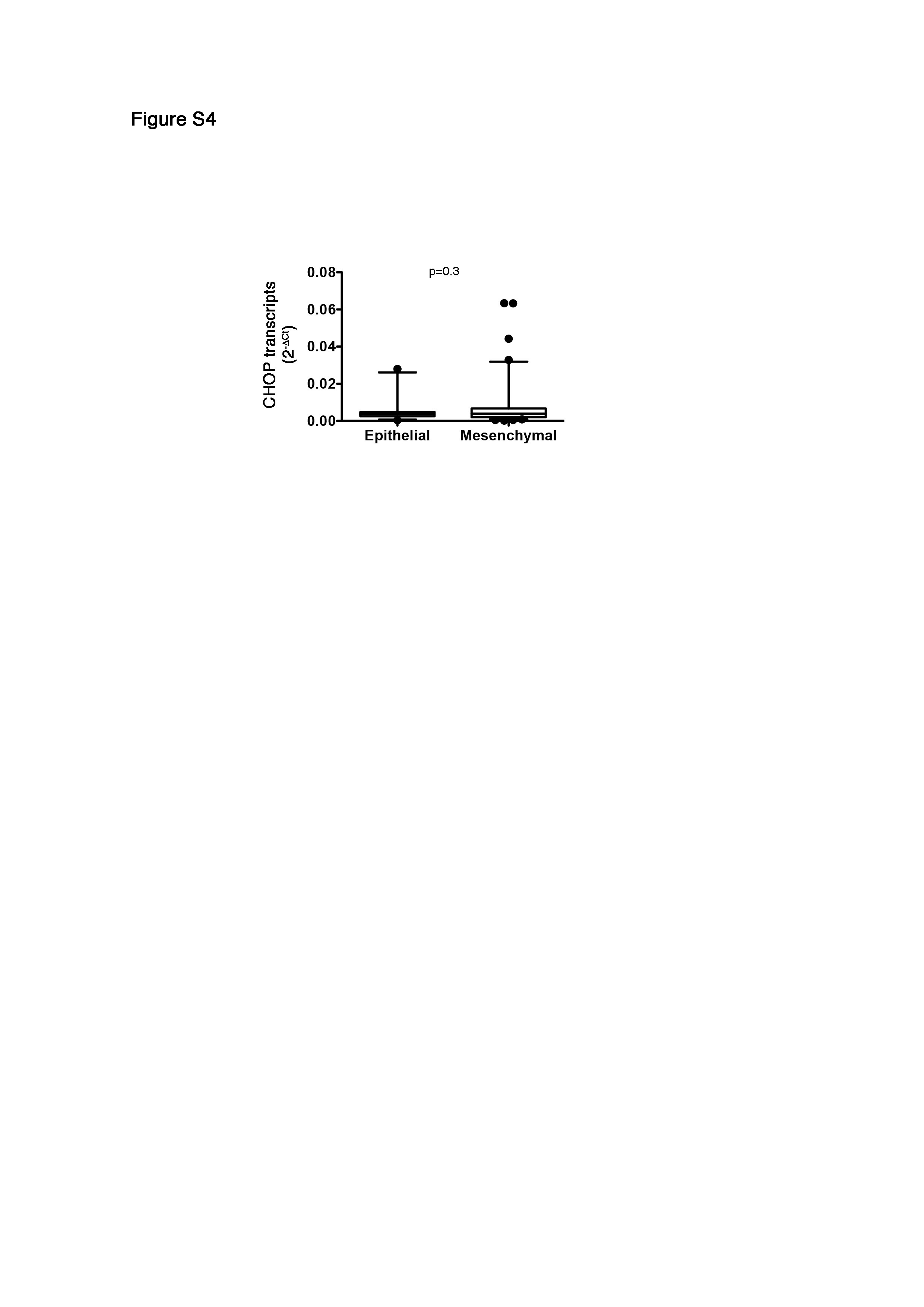

Supplement: Supplementary file 5 — Supplementary information5. [file 41598_2020_67243_MOESM5_ESM.tif]
